# Forest Trees in Human Modified Landscapes: Ecological and Genetic Drivers of Recruitment Failure in *Dysoxylum malabaricum* (Meliaceae)

**DOI:** 10.1371/journal.pone.0089437

**Published:** 2014-02-18

**Authors:** Sascha A. Ismail, Jaboury Ghazoul, Gudasalamani Ravikanth, Cheppudira G. Kushalappa, Ramanan Uma Shaanker, Chris J. Kettle

**Affiliations:** 1 ETH Zürich, Institute of Terrestrial Ecosystems, Ecosystem Management, Zürich, Switzerland; 2 Ashoka Trust for Research in Ecology and the Environment, Royal Enclave, Srirampura, Jakkur Post, Bangalore, India; 3 College of Forestry, University of Agricultural Sciences (Bangalore), Ponnampet, Kodagu district, Karnataka, India; 4 Department of Crop Physiology and School of Ecology and Conservation, University of Agricultural Sciences, GKVK Campus, Bangalore, India; National University of Singapore, Singapore

## Abstract

Tropical agro-forest landscapes are global priority areas for biodiversity conservation. Little is known about the ability of these landscapes to sustain large late successional forest trees upon which much forest biodiversity depends. These landscapes are subject to fragmentation and additional habitat degradation which may limit tree recruitment and thus compromise numerous ecosystem services including carbon storage and timber production. *Dysoxylum malabaricum* is a large canopy tree species in the Meliaceae, a family including many important tropical timber trees. This species is found in highly fragmented forest patches within a complex agro-forest landscape of the Western Ghats biodiversity hot spot, South India. In this paper we combined a molecular assessment of inbreeding with ecological and demographic data to explore the multiple threats to recruitment of this tree species. An evaluation of inbreeding, using eleven microsatellite loci in 297 nursery-reared seedlings collected form low and high density forest patches embedded in an agro-forest matrix, shows that mating between related individuals in low density patches leads to reduced seedling performance. By quantifying habitat degradation and tree recruitment within these forest patches we show that increasing canopy openness and the increased abundance of pioneer tree species lead to a general decline in the suitability of forest patches for the recruitment of *D. malabaricum*. We conclude that elevated inbreeding due to reduced adult tree density coupled with increased degradation of forest patches, limit the recruitment of this rare late successional tree species. Management strategies which maintain canopy cover and enhance local densities of adult trees in agro-forest mosaics will be required to ensure *D. malabaricum* persists in these landscapes. Our study highlights the need for a holistic understanding of the incipient processes that threaten populations of many important and rare tropical tree species in human dominated agro-forest landscapes.

## Introduction

Tropical forests are globally important centers of biological diversity and provide crucial ecosystem services including important terrestrial carbon stores [1]. The vast majority of tropical forest does not reside within protected areas [2] but rather as fragmented patches of forest within a more complex landscape mosaic. These forests are typically logged, degraded, highly patchy [3] and subject to a wide array of additional anthropogenic pressures such as grazing, hunting and invasive exotic species [4]. The persistence of late successional tree species within such human dominated tropical landscapes is important for maintaining tropical forest biodiversity. How well late successional tropical forest tree species are able to persist within these landscapes depends upon their resilience to these multiple and potentially synergistic genetic and ecological stressors [5], [6]. Few empirical studies have endeavored to evaluate these intrinsic genetic and extrinsic ecological stressors in unison despite these rapidly increasing pressures.

Studies investigating genetic consequences of fragmentation in woody species reveal that fragmentation can lead to elevated inbreeding [7], [8] and reduced fitness of the progeny [9], [10] through inbreeding depression. One possible underlying process is that pollinators predominantly forage among near neighbors, which may increase bi-parental inbreeding due to increased mating among few related individuals [11]. Habitat fragmentation has a number of ecological consequences for the reproductive ecology of plant species, for example by reducing pollinator visitation which directly reduces fruit and seed set [12]. Fragmentation can also have significant impacts on the community of seed dispersers influencing dispersal [13] and even on the selection pressures upon seed size [14]. When forests become fragmented, reproduction of many tree species can be altered due to unfavorable environmental conditions for seedling establishment. For example, tree seedling survival can be reduced due to drier conditions [15], increased litter fall, which smothers seedlings [16], and alien species invasion [17] especially at the forest edges. Recruitment of late successional shade tolerant trees is thought to be especially sensitive to fragmentation effects because of competition with faster growing light-demanding species that perform better under more open canopies [18]. Forest fragmentation may thus cause shifts in species compositions where slow growing late successional tree species are replaced by early successional pioneer species [19], [20].

Although genetic and non-genetic consequences of fragmentation have been shown to affect tree species reproductive ecology, these stressors are rarely considered in unison within a single study. Our objective was to empirically investigate if genetic and ecological processes affected recruitment of a late successional tree species within a fragmented landscape mosaic. We combined a molecular assessment of inbreeding in progeny of the canopy tree species *Dysoxylum malabaricum* with ecological and demographic data to address the question: can a late successional tropical tree species persist in a complex agro-forest landscape within the Western Ghats biodiversity hot spot, South India?

In a previous study we demonstrated that pollen dispersal of *D. malabaricum* in this fragmented landscape can be extensive. However, in the highly fragmented patches - when densities of adult trees were low (5 or fewer conspecific trees within 500 m) - the frequency of short distance pollen dispersal and consequently mating among related individuals was greater than in patches which were less fragmented (when local population densities were high, 6 or more conspecific trees within 500 m) [21]. The implications of this elevated inbreeding for growth and performance of progeny have until now not been examined. To evaluate the genetic and ecological factors which undermine the ability of *D. malabaricum* to persist in this landscape we present two data sets. The genetic data consists of a nursery trial of progeny collected from low and high density *D. malabaricum* forest patches. We used multilocus genotypes of these seedlings together with adult tree genotypes to determine the level of inbreeding and its implications for seedling vigour. The ecological data consists of detailed demographic data on the adult trees and densities of seedlings coupled with seven indicators of degradation in 17 forest patches, to investigate whether degradation correlated with seedling density. We used these data sets to test the following hypotheses: A) Mating between related individuals due to fragmentation reduces seedling performance in *D. malabaricum.* B) Ecological degradation leads to reduced *D. malabaricum* seedling densities in forest patches. By combining the two approaches above we hoped to gain a more holistic view of the factors influencing the demographic structure of *D. malabaricum* in this landscape. *D. malabaricum* is representative of numerous other threatened canopy tree species of late successional forest whose habitat is increasingly limited to forest patches within complex landscape mosaics. This study thus advances our understanding of how the genetic and ecological consequences of habitat fragmentation and degradation in unison undermine canopy tree persistence in human modified landscape mosaics.

## Methods

### Ethics statement

The forest patches we worked in are conserved due to cultural beliefs, owned by the Forest Department of Karnataka and managed by local temple committees, which granted access for sample collection. This was the only formal access permission required to conduct our research.

The study species *D. malabaricum* is not assessed under the IUCN Red List and is not listed as a protected species, but is classified as endangered under the Indian national threat assessment [22].

### Study area

**Figure 1:**
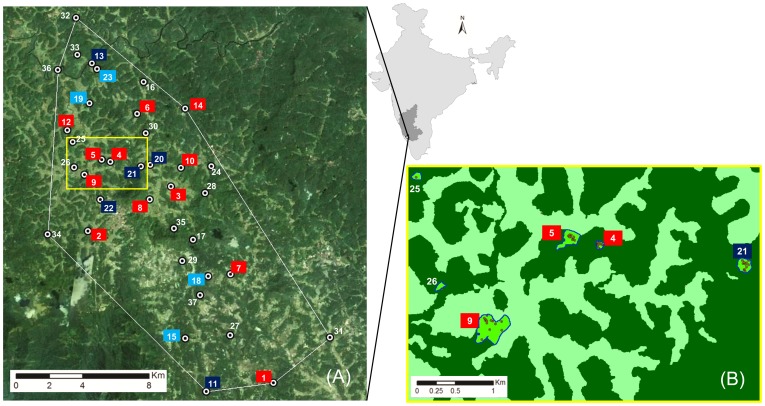
Location of the study area within India (light grey) and Karnataka (dark grey). Image (A): Study area marked with a white minimum convex polygon (216 km^2^) around the numbered forest patches where adult trees were found. The color labels of the numbers indicate if only *Dysoxylum malabaricum* seed for the nursery trial were collected (light blue), if only survey plots were established (dark blue) or if both records were taken (red). Image (B): Zoom of the yellow rectangle on image (A) with coffee plantations marked dark green and open areas (mainly paddy) marked light green. Investigated forest patches are the bright green polygons with a blue border and dots displaying adult *D. malabaricum* trees.

This study focusses on an agro-forest landscape encompassing 216 km^2^ including coffee, rice and forests patches (Figure 1). Our study site is situated in Kodagu district, within the Western Ghats biodiversity hotspot, South India. This district is a major coffee-growing region where coffee is grown predominantly under native shade trees [23]. Kodagu is renowned for the high density of small native forest patches conserved for cultural use [23]. These forest patches are set within an agricultural matrix consisting mainly of native shade coffee plantations and paddy fields [24]. Although these forest patches contribute only marginally to the overall forest area of the region [25], they are recognized as important repositories of biodiversity [26]. The forest patches within our study area are subject to anthropogenic disturbances through intensified resource extraction by the local community, such as fuel wood, small poles and non-timber forest products [27]. Illegal timber extraction and the encroachment of forest patches by coffee plantations are also common [28]. Within the landscape of the study area we have located and mapped all adult *D. malabaricum* trees [21].

### Study species

*Dysoxylum malabaricum* is a highly prized timber species within the tropical tree family Meliaceae. The species produces hermaphrodite flowers with a nectar reward suggesting insect pollination, but the main pollinators are unknown. Within the study area *D. malabaricum* maintains high genetic connectivity with around 9% of the pollen dispersal beyond 5 km distance [21]. The ripe fruit split along the four septa displaying the shiny brown seed (c. 30×20 mm) consumed by large-gape birds [29]. Our own field observations support the idea that the Malabar Grey Hornbill *Ocyceros griseus* is an important dispersal agent. The birds ingest the entire seed and remove the brown lipid rich seed coat before regurgitation. If the seed fall passively to the forest floor they rot quickly suggesting obligate zoochory (Ismail *pers. obs.* 2010). The Malabar Grey Hornbill is known to be relatively robust to habitat disturbances and is known to cross over open habitat [30] indicating the potential to disperse seed over long distances. The potential for long distance gene dispersal by pollen and seed is expected to buffer tree species against negative effects of fragmentation [31]. *D. malabaricum* has been extensively logged [32], and demand for *D. malabaricum* timber remains high with round wood logs fetching up to US$ 620 per cubic metre at local timber auctions in Kodagu (Ismail *pers. obs.* 2008).

Within our study area *D. malabaricum* is predominantly found in forest patches: of the 235 adult trees recorded, we found 223 trees in 35 forest patches. The remaining twelve trees were found in coffee plantations (of which nine were within 300 meters of a forest patch). Our previous study on pollen dispersal suggests that our sample of adult trees is exhaustive [21]. We have mapped, genotyped and recorded the diameter at breast height (DBH) of all adult *D. malabaricum* trees in our study area.

### Evaluation of seedling performance and inbreeding

**Nursery trials.** 617 seeds were collected near to 37 fruiting trees (97% of seed were found within 10 m of a fruiting tree) across 16 of the 35 forest patches within our study area (see Figure 1 and Table S1 in Supporting Information for details). Each seed was planted in a 1 liter polyethylene bag into a mixture of 1 part red soil, 2 parts cow dung and 2 parts river sand during July and August 2008, within 1 day after collection. A total of 363 seeds (61%) germinated. Of those that failed to germinate, at least 119 seeds (20%) were predated by larvae of a tephritid fly (*Dacus sp*.). Because of this initial exogenous loss we did not continue assessment of germination rates as a performance variable attributable to genetic factors. We grew the subsequent seedlings (n = 363) in a shade house (50% shade cloth) and watered daily. We rotated the seedlings monthly within the shade house to prevent any local effects, such as daylight orientation and neighbor competition. To reduce mortality due to pests we also applied insecticide and fungicide uniformly across the plants. Plants were repotted after 1 year into 5 liter polyethylene bags. Growth of seedlings in this controlled nursery experiment was monitored over 21 months by monthly measurements of total stem length from soil to top of the apical meristem, to an accuracy of 0.5 cm. After the 21 month period leaf material was sampled from each seedling for subsequent genotyping (see Appendix S1 in Supporting Information for details on genotyping and genetic analysis).

**Evaluation of seedling inbreeding.** To investigate if inbreeding and mating between related individuals reduced growth of the seedlings, we first applied a parentage analysis with the delta maximum-likelihood approach implemented in CERVUS [33] to determine the two most likely parents at the 90% confidence level (see Appendix S1 for details of the parameter settings). Candidate parents were all adult trees in our study area, genotypes of which had already been published in our earlier study [21]. The microsatellite data of the adult samples and the nursery seedlings were deposited in the DRYAD Digital Repository (doi:10.5061/dryad.3ck30 and doi:10.5061/dryad.mq8fn respectively).

The kinship coefficients [34] among the two assigned parent pairs provide one measure of inbreeding. In addition, individual inbreeding coefficients [35] were calculated for each seedling using the program SPAGEDI 1.3a [36] based on the seedling genotypes alone. The individual inbreeding coefficient ρ? is calculated as:
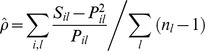



where P_il_ is the estimated frequency of the i_th_ allele at the l_th_ locus, S is an indicator variable which is one if the individual is homozygous for the i_th_ allele at the l_th_ locus and zero otherwise, and n_l_ is the number of alleles at the l_th_ locus.

**Characterizing local density of D. malabaricum adults in forest patches where seed was collected.** The 16 forest patches where we collected seed to investigate inbreeding effects were characterized as either low density (LD) or high density (HD) patches of *D. malabaricum* based upon the number of conspecifics within 500 m of any given adult tree within a patch following Ismail *et al.* [21]. This threshold of 500 m ensures that all conspecifics within the same forest patch are included and accounts for the rare cases where high local densities are the result of conspecific trees located in adjacent coffee plantations or in neighbouring forest patches [21]. This parameter reflects the local abundance of adult individuals of *D. malabaricum* and the distance to the neighboring forest patches and abundance of adult individuals within those neighboring patches. Only five of the 16 forest patches had another forest patch within 500 m and the distance between any two trees within a patch never exceeded 450 m. Our previous work using a detailed genetic analysis of pollen dispersal distances demonstrated increased genetic isolation and increased bi-parental inbreeding when local densities were below six conspecific trees within 500 meters [21] providing the justification for this categorization of genetic fragmentation.

**Statistical analysis of inbreeding and growth performance under nursery conditions.** Because the effect of local tree density in a forest patch has already been demonstrated to influence patterns of inbreeding in *D. malabaricum* [21], we classified the nursery seedlings based upon whether they were collected from low density (LD) or high density (HD) *D. malabaricum* forest patches. We tested for significant differences of median seedling height, median individual inbreeding coefficient and median kinship coefficient between seedlings of LD and HD origin with non-parametric Wilcoxon rank sum test implemented in R 2.13.1 [37]. To test for significant correlation of seedling height with individual inbreeding coefficients as well as with the kinship of parent pairs Pearson’s product-moment correlation (PPMC) test was computed in R [37]. Linear regression was not performed on this data due to variance inhomogeneity.

### Evaluation of recruitment and habitat degradation

**Sample plots.** To quantify habitat degradation and recruitment (indicated by seedling, sapling and pole stage tree densities) of *D. malabaricum* under natural conditions, we established five random plots in each of 17 selected forest patches (85 random plots) in 2010 (12 patches are common among both seed collection for inbreeding assessments and survey plots). For each patch we selected plots randomly in north orientated maps with an overlaid grid. Each grid cell was assigned a number, line by line from the top left to bottom right. We then used a random number generator in the program Excel (Microsoft) to select five random plots for each patch. The lower left-hand corner of the plot was defined by these coordinates. In case of overlap with other plots or when more than 10% of the plot was outside the forest patch the plot was turned 90° clockwise until the entire plot lay within the patch. Plots were 14 m×14 m except in the smallest forest patch where we had to reduce plot size to 10 m×10 m. We standardized all plot based measures to 100 m^2^. We quantified recruitment of *D. malabaricum* by recording the number of seedlings (< 50 cm height), number of saplings (> 50 cm, <150 cm) and poles (> 150 cm and DBH <5 cm) within each plot.

**Ecological parameters to quantify habitat degradation.** We recorded seven parameters which reflect the forest structure and species composition as indicators for habitat degradation. Within each plot this included three variables indicative of low habitat quality: 1) Canopy openness measured with a densiometer (model A) [38] (by averaging four measurements in the centre of the 7 m by 7 m quarters of each plot. In each quarter we measured in all four cardinal directions). 2) The number of coffee seedlings (< 150 cm height), because coffee *Coffea canephora* (a common and important exotic crop species) is naturalizing within forest patches of our study area (Ismail *pers. obs.* 2009). 3) Juvenile (> 0.25 m; <2 m) abundance of the pioneer tree species *Clerodendrum viscosum,* a light demanding species which dominates degraded forest. Four variables were selected as indicators of good habitat quality: 4) the number of adult *D. malabaricum* trees within a patch; 5) the total area of each forest patch (by mapping the forest patch with a handheld GPS (60CSx, Garmin, USA) to an accuracy of five meters). 6) The proportion of the forest patch border by shade coffee plantations. We chose this variable as shade coffee plantations of the region are predominately grown under dense native shade providing a more ‘forest like’ matrix than other land uses in the study area. This might buffer edge effects more effectively. 7) In addition we quantified the number of arboreal termite nests attached to branches and trees within each plot. Termites have been shown to be effective indicators of forest disturbances in other regions, because they are sensitive to forest disturbance, especially to canopy loss [39]–[41]. Using all these variables as indicators of habitat quality and *D. malabaricum* seedling density as an indicator of recruitment we tested the hypothesis that recruitment is reduced in degraded forest patches.

**Statistical analysis of recruitment and ecological degradation.** All statistical analysis was performed with R 2.13.1 [37]. To account for the nestedness of the study design we averaged the measurements of the five plots per forest patch before analysis. This resulted in 17 observations (for an overview of this data see Table S2). This approach was chosen as an alternative to a generalized linear mixed model based on the observations from all 85 plots, because non-normality and inhomogeneity of the residuals negated the use of these models. To investigate reproductive success of *D. malabaricum* in the wild, we performed multiple linear regression (MLR) models with seedling densities as the response variable and the seven variables on habitat degradation described above as explanatory variables. Model assumptions were checked for normality and homogeneity by visual inspections of plots of residuals against fitted values. To meet model assumptions seedling densities were square root transformed prior to analysis. On this initial model we applied model selection with the forward as well as the backward step function implemented in R to exclude irrelevant variables and to improve the model. Pearson’s product-moment correlation (PPMC) test was applied to check for co-linearity among pairs of explanatory variables.

## Results

### Effects of inbreeding and seedling performance

Of the 363 seeds which germinated 297 seedlings survived for 21 months. Using a parentage analysis we were able to assign 99% of seedlings at the 90 % confidence level to the two most likely parents (293 individuals). For subsequent analysis of growth performance 14 seedlings were excluded because the height measurements were ambiguous. This resulted in the exclusion of one of the forest patches from subsequent analysis of inbreeding. The classification of the remaining 273 seedlings into HD forest and LD forest provenance resulted in 197 seedlings from eight HD forest patches and 76 seedlings from seven LD forest patches.

**Figure 2:**
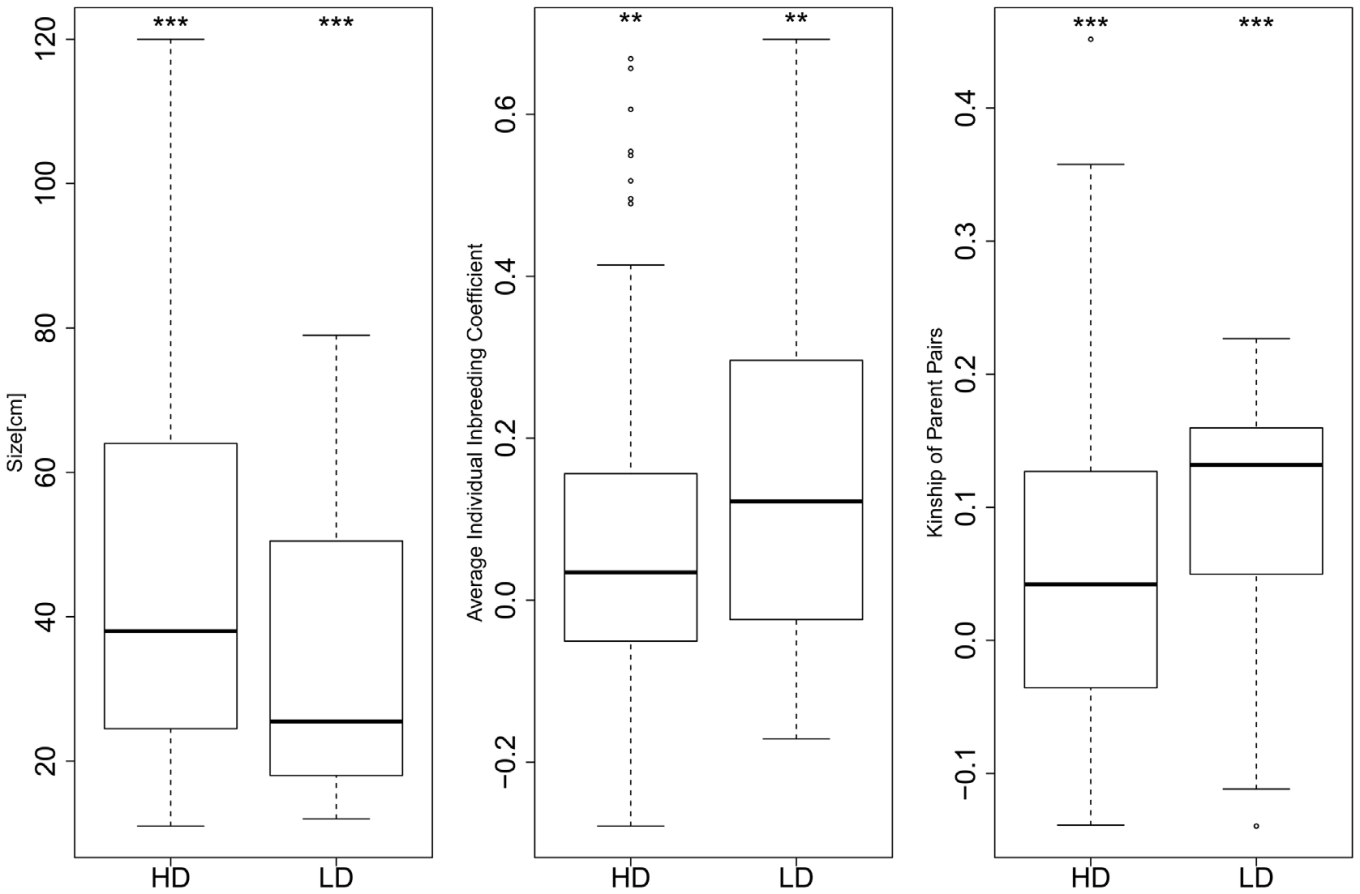
Effect of high density (HD) and low density (LD) of adult Dysoxylum malabaricum trees on A) seedling height after 21 months of growth (Median HD  =  38.0 cm, Median LD  =  25.5 cm), B) individual inbreeding coefficient (Median HD  =  0.034, Median LD  =  0.122), and C) pairwise parental kinship coefficients [32] (Median HD  =  0.042, Median LD  =  0.132), of nursery-reared D. malabaricum seedlings. Boxplots show the median and the upper and the lower quartile, the whiskers are 1.5 times the interquartile range from the box, dots outside of the whiskers are considered outliers. Significant differences are based on Wilcoxon rank sum test; * p<0.05, ** p<0.01, *** p<0.001.

Comparing the assigned seedlings originating from HD forest patches vs LD forest patches, the median ranks of the seedling height after 21 months were significantly higher (Median HD = 38.0 cm, Median LD  =  25.5 cm, p-value  =  0.00003), individual inbreeding coefficients and kinships of parent pairs were significantly lower (p-value  =  0.001 and p-value  =  0.0001 respectively) in seedlings from HD forest patches (Figure 2). Individual inbreeding coefficient was significantly negatively correlated with seedling height (PPMC coefficient  =  –0.139, t = –2.36, df = 281, p-value  =  0.02) (the scatterplot of this data is given in Figure S1). In contrast, we observed no significant correlation between kinship of parent pairs and height of the seedlings (t = –1.32, df = 271, p-value  = 0.19) (the scatterplot of this data is given in Figure S1).

### State of recruitment in *D. malabaricum*

**Figure 3:**
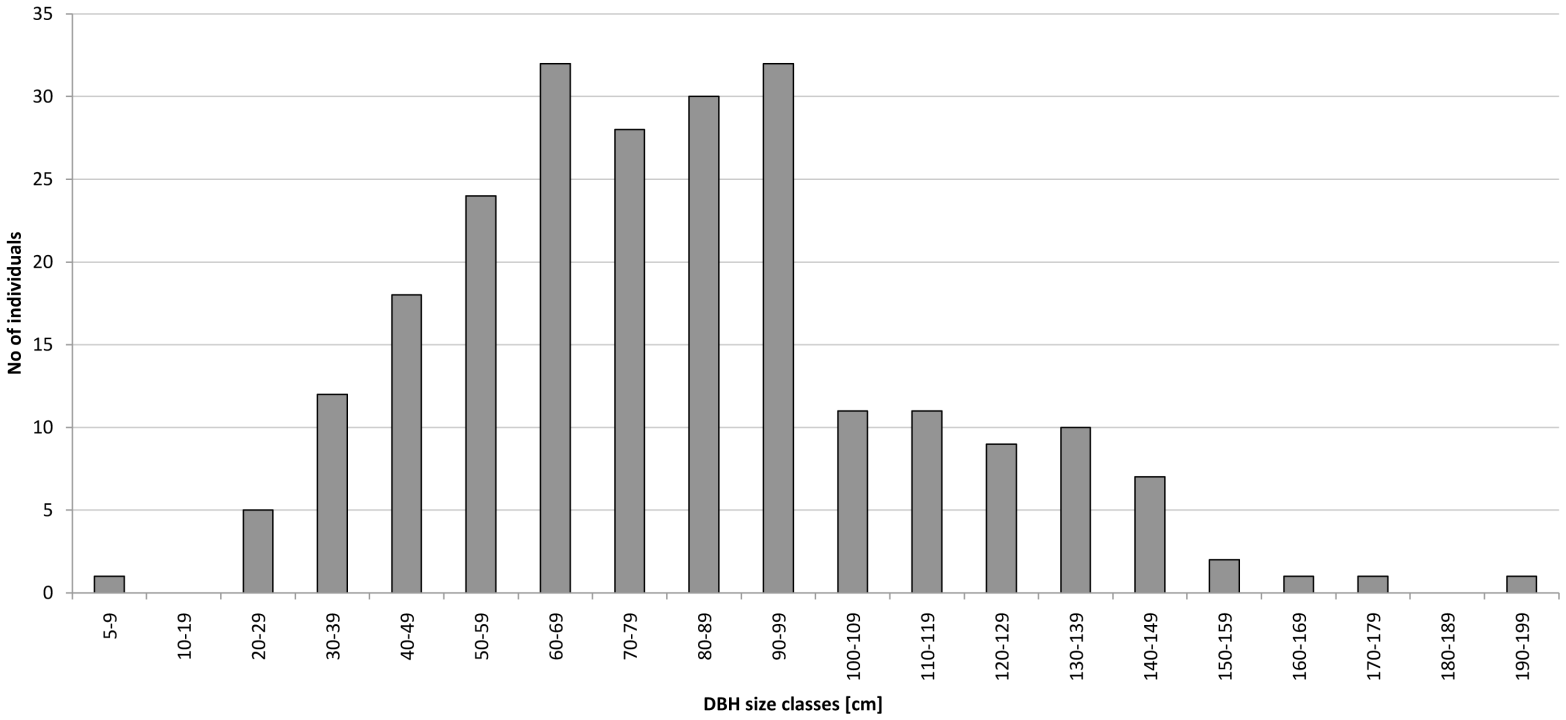
Histogram of diameter at breast height (DBH) of all the 235 enumerated adult Dysoxylum malabaricum trees within the 216 km^2^ study area.

Plotting size class frequency distributions of all recorded reproductive (> 5 cm DBH) *D. malabaricum* trees across the entire 216 km^2^ area showed an inverted ‘U’ shape distribution (Figure 3). There was a noticeable absence of the smallest size classes (< 20 cm DBH) with only one tree with a DBH of 8.5 cm and no trees in the 10 – 20 cm DBH class. There was a constant decline in the frequency of individuals from 60 cm DBH downward.

Although seedlings were present in all forest patches and saplings were present in nearly all surveyed forest patches, pole stage trees (> 1.5 m height and <5 cm DBH) were extremely rare. We detected only 11 poles in total in six of 17 surveyed forest patches. Seedling and sapling densities (averaged per site) varied greatly and ranged from 0.1 to 17.6 seedlings per 100 m^2^ and from 0 to 1.7 saplings per 100 m^2^. We only found pole stage trees at very low densities from 0 to 0.4 individuals per 100 m^2^.

### Effects of habitat degradation on recruitment of *D. malabaricum*

We used seven indicators of habitat degradation as explanatory variables of our MLR model to investigate their effect on seedling densities. In the model selection the number of adult *D. malabaricum* trees, the area of the forest patch and the density of coffee seedlings, did not improve the regression model and were excluded from the subsequent model. Within the final reduced model (Table 1) the percentage of closed canopy (t = 3.67, p = 0.0032) and the density of termite nests (t = 7.21, p = 0.00001) were positively associated with seedling density. The density of *C. viscosum* juveniles (t = –5.05, p = 0.0003) was negatively associated with seedling density. The proportion of the forest patch bordered by coffee plantations improved the model but was not significantly positively correlated with seedling density (t = 1.54, p = 0.149). PPMC among the pairs of explanatory variables was non-significant. The adjusted r squared of the final model was 0.81. Restricting the MLR only to the three significant variables resulted in an adjusted r squared of 0.79.

**Table 1:** Summary of the multiple linear regression after model selection used to fit *Dysoxylum malabricum* seedling densities under natural conditions.

	Estimate	Std. Error	t value	Pr(>|t|)	
(Intercept)	–35.007	9.796	–3.573	0.0038	**
% of closed canopy	0.375	0.102	3.671	0.0032	**
*C. viscosum* juveniles	–0.286	0.057	–5.053	0.0003	***
Termite nests	3.918	0.543	7.213	0.0000	***
% of border with coffee	0.008	0.005	1.542	0.1491	

* p<0.05, ** p<0.01, *** p<0.001.

## Discussion

The ability of late successional tropical tree species to persist in complex agro-forest landscapes will have significant implications for biodiversity and ecosystem services. Here we provide an example of one rare but important tropical tree *Dysoxylum malabaricum*. The nursery trial of genotyped seedlings indicates that fragmentation can cause reduced seedling vigour through inbreeding effects. Further, we demonstrate that habitat degradation reduces seedling densities, which appears to be associated with increased canopy disturbance. Thus fragmentation and degradation both appear to compromise the ability of this late successional tree to reproduce and persist in human modified landscapes. Below we examine the evidence for both genetic and ecological stressors influencing the recruitment of *D. malabaricum*. We discuss the implications of these findings for understanding of the long term persistence of late successional forest trees in tropical agro-forest landscapes.

### Importance of genetic factors for *D. malabaricum* recruitment

The present study, using nursery-grown seedlings and the kinship coefficients of their most likely parent pairs, confirms that increased inbreeding due to fragmentation reduces seedling growth. We interpret the reduced seedling average growth rates as a sign of inbreeding depression. Inbreeding depression has been shown to be a key factor reducing plant population viability [42] and is a plausible constraint to recruitment of *D. malabaricum.* Although alternative mechanisms for reduced performance exist, such as low pollen diversity [11] or maternal origin [9], the implications for reproduction success remain the same.

### Impact of habitat degradation on *D. malabaricum* recruitment

Our evaluation of habitat suitability suggests that degradation of forest patches as indicated by increased openness of the forest canopy is resulting in sites less favorable for *D. malabaricum* recruitment. This idea is supported by the negative association of *D. malabaricum* seedlings with the density of *C. viscosum* seedlings and the percentage of open canopy as well as the positive association with the density of arboreal termite nests, independent of adult tree abundance.

*Clerodendrum viscosum* is an early successional light demanding species establishing after gap formation [44]. We refrain from concluding a causal link between increased abundance of *C. vicosum* and lower *D. malabaricum* seedling densities. However, these associations are consistent with the idea that *D. malabaricum* seedlings in forest patches with more open canopy experienced increased competition from light demanding species, and a less favorable microclimate.

Interestingly, *D. malabaricum* seedling densities are greater where arboreal termite nests are more abundant, which could indicate more favorable and less degraded habitat. Termite communities were shown to be sensitive to canopy disturbance in other studies [39]–[41] which may well represent a response of termite communities to the loss of big trees. However, the processes underlying this correlation are likely to be more complicated, termites are also important agents of soil formation and nutrient cycling in tropical forests [45] and therefore may influence edaphic conditions important for *D. malabaricum* seedling establishment.

The additional factors we recorded (density of coffee seedlings, the number of adult *D. malabaricum* trees within a patch and the total area of each forest patch) were excluded by the model selection. However, it is noteworthy that though there was no statistically significant relationship between the number of coffee seedlings and *D. malabaricum* seedling densities, in plots where we observed high densities of coffee seedlings (> 200 per 100 m^2^) we consistently detected very low densities of *D. malabaricum* seedlings. These plots had on average more than six times lower *D. malabaricum* seedling densities than plots with lower coffee seedling densities (where we recorded very low to high densities of *D. malabaricum* seedlings*)*. The potential competitive interactions between *D. malabaricum* and coffee seedlings are worthy of future empirical validation. The observed variation of seedling densities across patches does not correspond with the density of reproductive adult trees and the area of the forest patches (no significant fits to the MLR) suggesting that seed production and suitable area for establishment *per se* are not driving the limited recruitment at the patch level.

### Evidence for recruitment limitation in *D. malabaricum*

Based upon estimates of seedling and sapling densities within our 85 random plots, seed production and germination appears to be substantial in *D. malabaricum*. However, evaluation of the population structure based upon tree diameter classes indicates an almost complete absence of the smallest size class (> 5 cm<20 cm DBH) trees across the entire 216 km^2^ study area (Figure 3). Based upon preliminary dendrological estimates in this species (see Appendix S2 in Supporting Information for details), we infer that trees greater than 20 cm DBH are at least 30 years old. We interpret this as a sign of poor recruitment from the sapling to the adult stage over the last 30 years. The survey plot data demonstrate a clear decline in abundance of adult trees from about 60 cm DBH to 20 cm DBH. This demographic structure is concurrent with a decline in recruitment since the intensification of commercial logging and plantation crops in the Western Ghats some 80– 100 years ago [43] and a lack of recruitment since the last extensive expansion of coffee plantations in the region during the last 30 years [23].

## Conclusions

Tropical agro-forest landscape mosaics offer great promise as habitats to support a wide array of tropical biodiversity [46] provided they retain key forest features such as diverse tree communities. Our analysis provides a general view of intrinsic genetic and extrinsic ecological factors which affect the recruitment of a rare late successional tropical tree species in forest fragments. *D. malabaricum* shows relatively long-distance pollen dispersal [21]. In addition, the seed disperser (the Malabar Grey Hornbill) moves in open habitat and can persist in fragmented forests [30]; thus one might predict that recruitment of *D. malabaricum* would be little affected by fragmentation and degradation [31]. However, this expectation was not supported by our study. Fragmentation and associated habitat degradation are likely to have direct but subtle genetic and ecological consequences for recruitment. Fragmentation can lead to elevated inbreeding which reduces seedling vigour. Degradation of remnant forest patches reduces the suitability for late successional tree species leading to a double-blow to recruitment. Because low levels of inbreeding might cause more pronounced fitness consequences under more stressful habitat conditions [47] the common co-occurrence of both genetic and ecological stressors might be highly relevant for forest dynamics in fragmented landscapes. Such multiple co-occurring ecological, genetic and anthropogenic stressors were shown to become disproportionally effective when local population densities become low (so called allee effects) [48]. These represent insidious processes, which in unison may undermine recruitment of late successional tree species. Considering the global pattern of late successional trees species being replaced by few pioneer tree species in tropical forest fragments [20], our study highlights the need for future research to recognise potentially interacting stressors which will advance our understanding of forest dynamics in mosaic landscapes in general. Conservation management therefore needs to address both the genetic and ecological stressors and consider that these stressors are likely to be amplified by low population densities.

A failure to conserve this important biodiversity within forest patches is likely to have implications for ecosystem processes and services. Indeed, it was shown that rare tree species contribute disproportionately to the maintenance of ecosystem processes [49]. Therefore a more holistic understanding of the threats to tree species in human modified landscapes, is urgently required if these landscapes are to sustain biodiversity and ecosystem services in the future.

## Supporting Information

Figure S1 **Scatterplots of** ***Dysoxylum malabricum*** **seedling height after 21 months of growth under nursery conditions against (A) individual inbreeding coefficient (Pearson product moment correlation coefficient  = −0.139, t** = **−2.36, df** = **281, p-value**  = **0.02) and (B) kinship of parent pairs (Pearson product moment correlation coefficient**  = **−0.079, t** = **−1.32, df** = **271, p-value**  = **0.19).**
(DOCX)

Appendix S1 **Methods of genotyping and genetic analysis.**
(DOCX)

Appendix S2 **Dendrological estimates.**
(DOCX)

Table S1 **Details of** ***Dysoxylum malabaricum*** **seed sampling.**
(DOCX)

Table S2 **Data on degradation and reproductive success of** ***Dysoxylum malabaricum*** **averaged per forest patch.**
(DOCX)
