# Supplementary material for: Forest Trees in Human Modified Landscapes: Ecological and Genetic Drivers of Recruitment Failure in Dysoxylum malabaricum (Meliaceae)
Source: PLoS One. 2014 Feb 18;9(2):e89437. doi: 10.1371/journal.pone.0089437 (PMC3928449; doi:10.1371/journal.pone.0089437)
Supplement: Figure S1 — Scatterplots of Dysoxylum malabricum seedling height after 21 months of growth under nursery conditions against (A) individual inbreeding coefficient (Pearson product moment correlation coefficient = −0.139, t = −2.36, df = 281, p-value = 0.02) and (B) kinship of parent pairs (Pearson product moment correlation coefficient = −0.079, t = −1.32, df = 271, p-value = 0.19). (DOCX) [file pone.0089437.s001.docx]

***
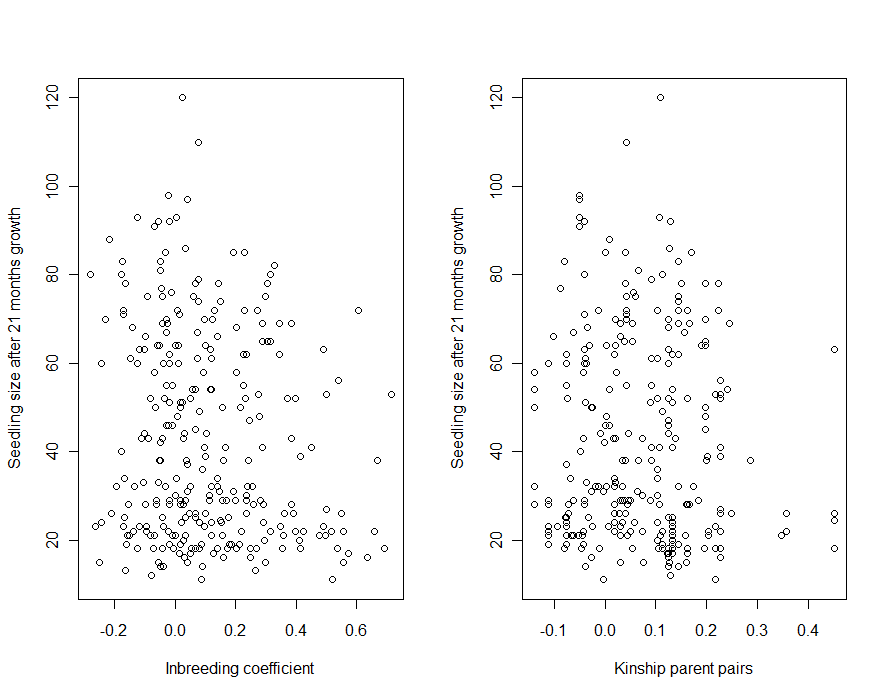
***

A

B

***Figure S1:*** Scatterplots of *Dysoxylum malabricum* seedling size after 21 months of growth under nursery conditions against (A) individual inbreeding coefficient (Pearson product moment correlation coefficient= -0.139, t = -2.36, df = 281, p-value = 0.02) and (B) pairwise kinship of parent pairs (Pearson product moment correlation coefficient= -0.079, t = -1.32, df = 271, p-value = 0.19).
