# Supplementary material for: Forest Trees in Human Modified Landscapes: Ecological and Genetic Drivers of Recruitment Failure in Dysoxylum malabaricum (Meliaceae)
Source: PLoS One. 2014 Feb 18;9(2):e89437. doi: 10.1371/journal.pone.0089437 (PMC3928449; doi:10.1371/journal.pone.0089437)
Supplement: Appendix S1 — Methods of genotyping and genetic analysis. (DOCX) [file pone.0089437.s002.docx]

***Appendix S1:*** Methods of genotyping and genetic analysis

After 21 months of growth measurements we collected leaf tissue from each plant, which was desiccated with silica gel prior to DNA extraction. DNA was extracted using a CTAB (cetyltrimethyl ammonium bromide) extraction (Sambrook *et al.* 1989). We genotyped 297 nursery raised seedlings at eleven polymorphic nuclear microsatellite markers using the primers Dysmal 01, Dysmal 02, Dysmal 03, Dysmal 07, Dysmal 09, Dysmal 13, Dysmal 14, Dysmal 17, Dysmal 18, Dysmal 22 and Dysmal 26 (Molecular Ecology Resources Primer Development *et al.* 2010). The polymerase chain reaction (PCR) conditions were as described in Ismail *et al.* 2012. Amplified fragments were analyzed on an ABI3730 (Applied Biosystems) capillary sequencer and scored relative to a LIZ500 HD size standard with GeneMapper 3.5 (Applied Biosystems). Over the 297 seedling samples we detected between 4 and 17 alleles per microsatellite locus and a total of 112 alleles with a proportion of 99.4% of loci genotyped. The genetic data of the 235 adult trees from 37 sites are taken from Ismail *et al.* 2012 where details of the sampling and genotyping are also described. To compare basic values of genetic diversity among the different cohorts (nursery samples, adults and seedling samples) we calculated observed and expected heterozygosity using GENEALEX 6.41 (Peakall and Smouse 2006). Multilocus allelic richness and inbreeding coefficients (Fis) were estimated with FSTAT 2.9.3.2 (Goudet 1995).

To detect the two most likely parents we applied parentage analysis with the delta maximum-likelihood approach implemented in CERVUS (Marshall *et al.* 1998).

We ran simulations of parentage based on the allele frequencies of the adult trees with the following parameters: 10 000 simulated offspring with all 235 adult trees as candidate parents, estimated to represent 98% of the parents within the area (allowing for selfing). The minimum of matching loci was set at six; the error rate of mistyped loci was set 0.01 and the proportion of loci genotyped was 0.98. The significance threshold for the assignment of the two most likely parents was set at 90%.

**References**

Goudet, J. (1995). FSTAT (Version 1.2): A computer program to calculate F-statistics. *Journal of Heredity* **86**(6), 485-486.

Ismail, S. A., J. Ghazoul, G. Ravikanth, R. Uma Shaanker, C. G. Kushalappa and C. J. Kettle (2012). Does long-distance pollen dispersal preclude inbreeding in tropical trees? Fragmentation genetics of Dysoxylum malabaricum in an agro-forest landscape. *Molecular Ecology* **21**(22), 5484-5496.

Marshall, T. C., J. Slate, L. E. B. Kruuk and J. M. Pemberton (1998). Statistical confidence for likelihood-based paternity inference in natural populations. *Molecular Ecology* **7**(5), 639-655.

Molecular Ecology Resources Primer Development, C., J. An, A. Bechet, Å. Berggren, S. K. Brown, M. W. Bruford, Q. Cai, A. Cassel-Lundhagen, F. Cezilly, S.-L. Chen, W. E. I. Cheng, S.-K. Choi*, et al.* (2010). Permanent Genetic Resources added to Molecular Ecology Resources Database 1 October 2009–30 November 2009. *Molecular Ecology Resources* **10**(2), 404-408.

Peakall, R. and P. E. Smouse (2006). GENALEX 6: genetic analysis in Excel. Population genetic software for teaching and research. *Molecular Ecology Notes* **6**(1), 288-295.

Sambrook, J., E. F. Fritsch and T. Maniattis (1989). Molecular cloning: A laboratory manual, 2^nd^ ed. New York, Cold Spring Harbor Laboratory Press.
