# Supplementary material for: Forest Trees in Human Modified Landscapes: Ecological and Genetic Drivers of Recruitment Failure in Dysoxylum malabaricum (Meliaceae)
Source: PLoS One. 2014 Feb 18;9(2):e89437. doi: 10.1371/journal.pone.0089437 (PMC3928449; doi:10.1371/journal.pone.0089437)
Supplement: Appendix S2 — Dendrological estimates. (DOCX) [file pone.0089437.s003.docx]

***Appendix S2:*** Dendrological estimates

Although age estimates of tropical trees are difficult we approximate the larger size class trees (DBH 60-100 cm) of adult *D. malabaricum* trees in our study area to be between 100 to 160 years old. This is based upon tree ring counts of two felled *D. malabaricum* trees of 88cm and 104cm DBH which had 244 rings and 332 rings respectively. Because tree rings in tropical trees are rarely strictly annual (Worbes 2002) and because yearly rings would result in improbable old ages we assume a maximum of two rings per year (one during the monsoon season and one during the drought season). Given the strict seasonality in the region this assumption seems plausible because both long periods of inundation and drought produce tree rings in tropical trees (Worbes 2002). We estimate a diameter growth rate of approximately 3- 4 mm per year which is in the range of the highest published growth rates for the same region (Rai 1981). This approximation thus represents a minimum age estimate.

**References**

Rai, S. (1981). Rate of growth of some evergreen species. *Indian Forester* **107**(8), 513-518.

Worbes, M. (2002). One hundred years of tree-ring research in the tropics–a brief history and an outlook to future challenges. *Dendrochronologia* **20**(1), 217-231.
