# Supplementary material for: Forest Trees in Human Modified Landscapes: Ecological and Genetic Drivers of Recruitment Failure in Dysoxylum malabaricum (Meliaceae)
Source: PLoS One. 2014 Feb 18;9(2):e89437. doi: 10.1371/journal.pone.0089437 (PMC3928449; doi:10.1371/journal.pone.0089437)
Supplement: Table S1 — Details of Dysoxylum malabaricum seed sampling. (DOCX) [file pone.0089437.s004.docx]

***Table S1:*** Details of *Dysoxylum malabaricum* seed sampling for the nursery experiment.

| **Site ID** | **Total seed planted per site** | **Total survived 21 months per site** | **ID of nearest adult tree** | **Total seed planted per adult tree** | **Total survived 21 months per adult tree** |
| --- | --- | --- | --- | --- | --- |
| 23 | 20 | 1 | Dm374 | 20 | 1 |
| 1 |  |  | Dm94 | 13 | 11 |
| 1 | 17 | 15 | Dm68a | 4 | 4 |
| 2 |  |  | Dm325 | 8 | 4 |
| 2 |  |  | Dm332 | 21 | 12 |
| 2 |  |  | Dm333 | 9 | 4 |
| 2 | 58 | 26 | Dm329 | 20 | 6 |
| 3 |  |  | Dm184 | 20 | 19 |
| 3 | 40 | 27 | Dm180 | 20 | 8 |
| 4 |  |  | Dm390 | 20 | 9 |
| 4 |  |  | Dm393 | 29 | 12 |
| 4 | 81 | 31 | Dm386 | 32 | 10 |
| 5 |  |  | Dm376 | 20 | 10 |
| 5 |  |  | Dm377 | 20 | 11 |
| 5 | 60 | 35 | Dm383 | 20 | 14 |
| 6 |  |  | Dm158 | 20 | 4 |
| 6 | 21 | 5 | Dm158 | 1 | 1 |
| 7 |  |  | Dm70 | 11 | 9 |
| 7 |  |  | Dm71 | 10 | 5 |
| 7 |  |  | Dm149 | 21 | 14 |
| 7 |  |  | Dm150far | 5 | 4 |
| 7 | 67 | 36 | Dm26 | 20 | 4 |
| 8 |  |  | Dm186 | 20 | 7 |
| 8 |  | 25 | Dm186far | 20 | 14 |
| 8 | 45 |  | Dm185 | 5 | 4 |
| 9 |  |  | Dm49 | 20 | 1 |
| 9 |  |  | Dm227 | 20 | 9 |
| 9 |  |  | Dm226 | 18 | 4 |
| 9 | 66 | 15 | Dm312 | 8 | 1 |
| 10 |  |  | Dm503 | 18 | 6 |
| 10 | 24 | 6 | Dm504 | 6 | 0 |
| 18 |  |  | Dm400 | 25 | 16 |
| 18 |  |  | Dm369 | 42 | 31 |
| 18 | 74 | 51 | Dm398 | 7 | 4 |
| 19 | 3 | 2 | Dm159 | 3 | 2 |
| 12 | 1 | 1 | Dm47 | 1 | 1 |
| 14 | 20 | 2 | Dm373 | 20 | 2 |
| 15 | 20 | 19 | Dm365 | 20 | 19 |
| **Sum** | **617** | **297** |  | **617** | **297** |
