# Supplementary material for: Forest Trees in Human Modified Landscapes: Ecological and Genetic Drivers of Recruitment Failure in Dysoxylum malabaricum (Meliaceae)
Source: PLoS One. 2014 Feb 18;9(2):e89437. doi: 10.1371/journal.pone.0089437 (PMC3928449; doi:10.1371/journal.pone.0089437)
Supplement: Table S2 — Data on degradation and reproductive success of Dysoxylum malabaricum averaged per forest patch. (DOCX) [file pone.0089437.s005.docx]

***Table S2:*** Data on degradation and reproductive success of *Dysoxylum malabaricum* averaged per forest patch.

| **Forest patch ID** | **Dm seedling density** | **Dm sapling density** | **Dm pole density** | **Canopy closure %** | **Coffee seedling density** | **Cv juvenile density** | **Number of adult trees per forest patch** | **Forest patch area [ha]** | **% border with shade coffee plantation** | **Arboreal termite nest density** |
| --- | --- | --- | --- | --- | --- | --- | --- | --- | --- | --- |
| 12 | 1.0 | 0.2 | 0.4 | 96.1 | 295.2 | 6.2 | 1 | 0.8 | 100 | 0.2 |
| 1 | 2.2 | 1.1 | 0.2 | 93.8 | 143.3 | 0.0 | 9 | 3.0 | 60 | 0.2 |
| 3 | 17.6 | 0.6 | 0.4 | 94.7 | 8.9 | 1.0 | 5 | 2.4 | 83 | 0.8 |
| 5 | 0.7 | 0.9 | 0.0 | 94.3 | 72.7 | 2.7 | 13 | 4.5 | 79 | 0.2 |
| 4 | 0.7 | 0.1 | 0.0 | 95.8 | 353.7 | 3.3 | 12 | 1.0 | 100 | 0.0 |
| 20 | 1.0 | 0.5 | 0.0 | 95.8 | 10.9 | 1.4 | 10 | 2.2 | 67 | 0.1 |
| 21 | 0.3 | 0.5 | 0.0 | 94.0 | 161.2 | 1.3 | 11 | 3.2 | 100 | 0.0 |
| 7 | 0.1 | 0.0 | 0.0 | 93.0 | 265.4 | 3.3 | 11 | 7.5 | 93 | 0.2 |
| 8 | 6.4 | 0.3 | 0.0 | 97.1 | 44.4 | 0.0 | 4 | 2.0 | 33 | 0.3 |
| 10 | 0.4 | 0.3 | 0.1 | 94.0 | 239.3 | 0.4 | 3 | 1.6 | 87 | 0.1 |
| 22 | 3.7 | 0.2 | 0.0 | 95.5 | 172.8 | 1.0 | 11 | 2.8 | 86 | 0.0 |
| 14 | 0.2 | 0.1 | 0.0 | 96.2 | 228.7 | 3.8 | 4 | 5.1 | 100 | 0.1 |
| 9 | 3.7 | 1.7 | 0.1 | 95.8 | 99.5 | 0.2 | 15 | 14.4 | 51 | 0.0 |
| 11 | 1.9 | 0.0 | 0.0 | 95.6 | 16.0 | 7.3 | 7 | 1.5 | 100 | 0.4 |
| 6 | 0.6 | 0.3 | 0.0 | 92.8 | 157.8 | 0.0 | 16 | 8.0 | 60 | 0.1 |
| 2 | 0.1 | 0.1 | 0.0 | 96.6 | 57.1 | 5.8 | 30 | 8.8 | 25 | 0.1 |
| 13 | 0.1 | 0.0 | 0.1 | 94.5 | 58.6 | 1.5 | 1 | 3.9 | 52 | 0.0 |

All figures were averaged across 5 random plots per forest patch except: Number of adult trees per forest patch, Forest patch area [ha] and % border with shade coffee plantation
Dm = *Dysoxylum malabaricum*Cv = *Clerodendrum viscosum*
